# Evidence for a model of conformational change by the *Plasmodium falciparum* circumsporozoite protein during sporozoite development in the mosquito host through the use of camelid single-domain antibodies

**DOI:** 10.1128/iai.00081-25

**Published:** 2025-04-28

**Authors:** Rob Geens, Line De Vocht, Manuela C. Aguirre-Botero, Cécile Vincke, Ema Romão, Stefan Magez, Serge Muyldermans, Rogerio Amino, Yann G.-J. Sterckx

**Affiliations:** 1 Laboratory of Medical Biochemistry (LMB) and the Infla-Med Centre of Excellence, Department of Pharmaceutical Sciences, University of Antwerp 26660, Antwerp, Belgium; 2 Institut Pasteur, Université Paris Cité, Malaria Infection & Immunity 27058, Paris, Île-de-France, France; 3 Lab of Cellular and Molecular Immunology (CMIM), Brussels Center for Immunology (BCIM), Department of Bioengineering Sciences, Vrije Universiteit Brussel 70493, Brussels, Belgium; 4 Myeloid Cell Immunology Lab, VIB Center for Inflammation Research, Brussels, Belgium; University of California Davis, Davis, California, USA

**Keywords:** malaria, *Plasmodium falciparum*, sporozoite, circumsporozoite protein, camelid single-domain antibodies

## Abstract

*Plasmodium* sporozoites (SPZs) are formed in the *Anopheles* mosquito midgut from where they travel to the salivary glands and subsequently to the mammalian liver after deposition into the skin. The SPZ’s main surface antigen, the circumsporozoite protein (CSP), plays a pivotal role in SPZ biology and constitutes the immunodominant target for host antibodies. In this study, we raised single-domain antibodies (sdAbs) against CSP from *P. falciparum* (PfCSP) by immunizing two alpacas with recombinant versions of the antigen. We found that all identified sdAbs specifically target PfCSP’s globular αTSR domain without cross-reacting with *P. berghei* CSP. Further characterization revealed that most sdAbs recognize native PfCSP on the SPZ surface, although they do not have any inhibitory effect on hepatocyte binding and invasion. Structural studies showed that all binders target the previously identified α-epitope, confirming the non-protective nature of this epitope. Comparison of sdAb binding to midgut and salivary gland SPZs revealed a shift in the exposure and accessibility of the α-epitope. Hence, our findings provide further evidence that CSP undergoes structural changes during SPZ development in the mosquito host.

## INTRODUCTION

Malaria is a devastating disease that presents an immediate health threat for almost half the world’s population, with an estimated 263 million cases and 597,000 deaths in 2023 (1). The endemic ‘hot spot’ is Sub-Saharan Africa, which accounts for 94 and 95% of all malaria cases and deaths, respectively. The disease is caused by parasites of the *Plasmodium* genus and vectored by female *Anopheles* mosquitoes. The parasite sexually reproduces in the mosquito midgut and generates thousands of sporozoites (SPZs), which travel to the salivary glands where they await injection into the skin of the mammalian host. From their inoculation site, the SPZs travel to the liver where they differentiate into tens of thousands of merozoites. The latter re-emerge in the circulation to replicate within erythrocytes and cause the notorious malaria pathology (2). Most human malaria cases concern infections by *P. falciparum*, which causes the deadliest malaria. Due to the fast emergence of resistance against antimalarial drugs and insecticides (3, 4), intensive research has been performed during the past decades in the quest to design an effective vaccine (5). Recently, the first malaria vaccines have been approved (Mosquirix, also known as RTS,S/AS01, and R21/Matrix-M), which are subunit vaccines based on the *P. falciparum* circumsporozoite protein (PfCSP) (6, 7).

The choice for CSP as the basis for subunit vaccines is well considered, as it is an immunodominant SPZ surface antigen (8). Furthermore, CSP plays a central role during the SPZ life stage both in insect and mammalian hosts (9), where it has been reported to: (i) be essential for SPZ development in the mosquito (10, 11); (ii) form a shield around the SPZ to protect its plasma membrane against pore-forming proteins during its migration through the skin (12); (iii) be essential for the recognition and invasion of host hepatocytes via its interaction with highly sulfated heparan sulphate proteoglycans (HS-HSPGs) (13, 14); and (iv) function as extra- and intracellular effector biomolecules to dampen host inflammatory responses (15, 16). PfCSP contains an intrinsically disordered N-terminal domain (PfCSP_N_) (17), a flexible linker region composed of NANP and NVDP repeats (PfCSP_rep_) and is flanked by N- and C-terminal junctions (PfCSP_NTJ_ and PfCSP_CTJ_, respectively) and a globular C-terminal domain (PfCSP_C_) adopting a so-called αTSR fold (18) that is connected to the SPZ plasma membrane via a GPI anchor (19). Most antibodies (Abs) elicited by natural exposure or immunization target PfCSP_rep_ and are regarded as potent inhibitors of SPZ migration and invasion (12, 20–22). Importantly, despite the immunodominance of PfCSP_rep_, Abs targeting other CSP regions have been reported as well and may or may not be protective (23). Abs targeting PfCSP_N_ are rare and generally bind within or close to the biologically important Region I. However, their protective efficacy is still heavily debated (24–27). Abs recognizing PfCSP_NTJ_ have also been discovered and are currently considered the most potent inhibitory Ab type with regard to parasite development (28, 29). Finally, Abs targeting PfCSP_C_ are relatively rare and so far found to have poor SPZ inhibitory efficacy (30–32). However, it remains unclear whether the targeted epitopes are truly non-protective or whether the Abs are unable to reach their target because of the dense CSP packing on the SPZ surface.

The limited accessibility of CSP_C_ epitopes may be explained by the model proposed by Coppi and colleagues (33). Here, CSP is suggested to adopt two conformational states that are timely regulated to address the SPZ’s needs at different stages: the αTSR-exposed (or adhesive) conformation for midgut SPZs (MG SPZs) and the αTSR-shielded (or non-adhesive) conformation for salivary gland SPZs (SG SPZs). Since the αTSR domain is presumed to be involved in high-affinity cell adhesion processes but does not selectively target hepatocytes, it is proposed to be masked by CSP_N_ during the SPZ’s journey from the mosquito midgut to the mammalian liver. Hence, the different CSP conformations are believed to be linked to SPZ migration/invasion, in which the adhesive conformation only occurs at the start (development in the mosquito midgut) and end (hepatocyte invasion) of the SPZ life stage. Regarding the latter, the transition to the adhesive form is induced by interactions between CSP_N_ and HS-HSPGs presented on liver cells (13, 14), which eventually result in the proteolytic cleavage of CSP_N_ (34). This is presumed to ensure that CSP_C_ is only exposed just before hepatocyte invasion, thereby limiting possible interference by anti-CSP_C_ Abs. Although the Coppi model is based on *P. berghei* SPZs expressing wild-type or mutant PbCSP on their surface, another study has shown that similar mechanisms are involved during a *P. falciparum* infection (25).

In this paper, we describe the functional characterization of 12 camelid single-domain antibodies (sdAbs, also known as nanobodies) targeting PfCSP_C_. sdAbs correspond to the variable domain (VHH) of heavy-chain only Abs (HCAbs) found in camelids (llamas, alpacas, camels, and dromedaries) (35, 36). Two immune libraries were generated after the immunization of two alpacas with distinct PfCSP constructs, resulting in the identification of 12 specific sdAbs. Most binders have no effect on hepatocyte binding and invasion by SG SPZs. A structural characterization of the sdAb-PfCSP_C_ complexes through macromolecular X-ray crystallography (MX) and/or AlphaFold-Multimer shows that most sdAbs target the non-protective α-epitope (30–32). Interestingly, prominent sdAb binding signals could be observed on MG SPZs, thereby revealing that the α-epitope displays greater accessibility on MG SPZs compared to SG SPZs. Hence, our findings support that CSP is subject to conformational change during SPZ development in the mosquito.

## MATERIALS AND METHODS

### Recombinant production and purification of PfCSP_FL_ and PfCSP_C_

The *PfCSP_FL_*, *PfCSP_C_*, and *PbCSP_FL_* genes were synthesized, codon-optimized for bacterial expression, and cloned into the pET-21b(+) vector through the *Nde*I and *Xho*I sites via a commercial partner (GenScript). The PfCSP_FL_ and PfCSP_C_ constructs are based on *P. falciparum* NF54 CSP (Uniprot ID: P19597) and correspond to full-length CSP (F20-S384, devoid of signal peptide and GPI anchor) and its C-terminal domain (E310-S384), respectively. PbCSP_FL_ corresponds to full-length *P. berghei* ANKA CSP (NCBI ID XP_022712148.1, G21-S327, devoid of signal peptide and GPI anchor). All constructs contain a C-terminal TEV cleavage site (ENLYFQSGG) and a hexahistidine (His_6_)-tag. Chemocompetent *Escherichia coli* SHuffle T7 Express cells (NEB) were heat-transformed for gene expression, and transformants were selected on LB-agar-Gluc-Amp plates (LB-agar plates supplemented with 2% D-glucose and 100 µg/mL ampicillin) incubated overnight at 37°C. Pre-cultures of 10–25 mL were started by inoculation of 2xTY-Gluc-Amp medium (2xTY medium at pH 8.2 supplemented with 0.2% D-glucose and 100 µg/mL ampicillin) with a single colony or cell paste from a glycerol stock and grown overnight, shaking at 37°C. Main cultures of 0.5–1 L were inoculated with a 250-fold dilution of pre-culture and grown, shaking at 37°C to an OD_600_ of 0.6–0.9, at which gene expression was induced with 1 mM isopropyl β-D-1-thiogalactopyranoside (IPTG), and the temperature was decreased to 20°C for overnight incubation. Cells were harvested 20 h post-induction by centrifugation (3,220 ×*g* for 30 min at 4°C). Cell pellets were resuspended in lysis buffer (50 mM Tris–HCl, 500 mM NaCl, 20 mM imidazole, 2 µM leupeptin, 125 µM AEBSF, 100 µM EDTA, pH 8.0), flash frozen in liquid nitrogen, and stored at −20°C. A biotinylated PfCSP_FL_ construct (PfCSP_FL-bio_) was designed by inserting an AVI-tag (GLNDIFEAQKIEWHEGS) in between the TEV cleavage site and the His_6_-tag for site-specific biotinylation with *E. coli* biotin ligase (BirA). Chemocompetent *E. coli* SHuffle T7 cells (NEB) were co-transformed with PfCSP_FL-bio_ and BirA encoding (pBirAcm, Avidity) plasmids for *in vivo* production and biotinylation in 2xTY-Gluc-Amp-Chl medium (2xTY-Gluc-Amp medium supplemented with 35 µg/mL chloramphenicol) according to the procedures described above. The medium was supplemented with 50 µM D-biotin 30 min before induction.

The recombinant proteins were purified through immobilized metal affinity and size exclusion chromatography (IMAC and SEC, respectively). The purification protocol for PfCSP_FL_/PfCSP_FL-bio_/PbCSP_FL_ contains an additional heparin affinity chromatography (HAC) step to ensure their functional and biological relevance (18). Thawed cells were lysed by ultrasonication (60 cycles of 5 s pulses at 20% amplitude output and 5 s breaks on Sonics VCX-130) on ice, after which the soluble fraction was obtained by centrifugation (20,000 ×*g* for 1 h at 4°C). The cell extract (supernatant) was filtered (0.45 µm filter) prior to loading it onto a 5 mL HisTRAP HP column (Cytiva), pre-equilibrated in IMAC buffer A (50 mM Tris–HCl, 500 mM NaCl, 20 mM imidazole, 2 µM leupeptin, 125 µM AEBSF, 100 µM EDTA, pH 8.0). Contaminants were removed by washing the column with 10 column volumes (CVs) IMAC buffer A, and target protein was eluted by linearly increasing the imidazole concentration with IMAC buffer B (50 mM Tris–HCl, 500 mM NaCl, 500 mM imidazole, 2 µM leupeptin, 125 µM AEBSF, 100 µM EDTA, pH 8.0) from 0 to 100%. Relevant IMAC elution fractions containing the target protein were pooled and dialyzed overnight at 4°C against SEC running buffer (50 mM Tris–HCl, 500 mM NaCl, 2 µM leupeptin, 125 µM AEBSF, 100 µM EDTA, pH 8.0) to reduce the imidazole concentration. The dialyzed sample was concentrated using a Vivaspin Turbo 15 PES (10 or 5 k) concentrator device (Sartorius) and subjected to SEC on a pre-equilibrated HiLoad 16/600 Superdex 200 column (Cytiva) or a HiLoad 16/60 Superdex 75 column (Cytiva) for PfCSP_FL_/PfCSP_FL-bio_/PbCSP_FL_ and PfCSP_C_, respectively. For PfCSP_C_, relevant SEC elution fractions containing the target protein were pooled and stored at 4°C. For PfCSP_FL_/PfCSP_FL-bio_/PbCSP_FL_, relevant SEC elution fractions containing the target protein were pooled and dialyzed overnight at 4°C against HAC buffer A (50 mM Tris–HCl, 2 µM leupeptin, 125 µM AEBSF, 100 µM EDTA, pH 8.0) to reduce the salt concentration. The dialyzed sample was loaded onto a 1 mL HiTRAP Heparin HP column (Cytiva) pre-equilibrated with HAC buffer A. Remaining contaminants and non-functional PfCSP_FL_/PfCSP_FL-bio_/PbCSP_FL_ were removed by washing the column with 5 CVs HAC buffer A, and functional target protein was eluted by linearly increasing the salt concentration with HAC buffer B (50 mM Tris–HCl, 1 M NaCl, 2 µM leupeptin, 125 µM AEBSF, 100 µM EDTA, pH 8.0) from 0 to 100%. Fractions containing the target protein were pooled and stored at 4°C. Purity was analyzed by SDS-PAGE and anti-His western blotting, and biotinylation of PfCSP_FL-bio_ was verified by streptavidin western blotting.

### Generation of anti-PfCSP camelid single-domain antibodies

*Ethical statement*. All animal experiments were carried out according to the directive 2010/63/EU of the European Parliament for the protection of animals used for scientific purposes and approved by the Ethical Committee for Animal Experiments of the Vrije Universiteit Brussel (clearance number 14-220-19 for the alpaca immunization).

*Construction of anti-PfCSP immune libraries*. Two alpacas named Attitude and Cosimo were immunized with PfCSP_FL_ and PfCSP_C_, respectively, over a course of 6 weeks. Every week, 100 µg recombinant protein prepared in a volume of 500 µL was mixed with an equal volume of GERBU adjuvant (GERBU Biotechnik GmbH, Germany) prior to injection. A total of 100 mL of blood was collected from each alpaca 4 days after the last immunization. The two immune libraries (one for each alpaca) were constructed as described previously (37).

*Phage display and panning of the anti-PfCSP libraries*. Enrichment and identification of PfCSP-specific sdAbs from the immune libraries were performed according to published procedures (38). The two libraries (100 µL) were grown, shaking in 100 mL 2xTY-Gluc-Amp medium (1% D-glucose) at 37°C to an OD_600_ of 0.6–0.8. After that, they were infected with 10^12^ M13K07 helper phages (Invitrogen) for 30 min at room temperature. Cells were harvested by centrifugation (1,500 ×*g* for 10 min at 4°C), inoculated in 300 mL 2xTY-Amp-Kan medium (2xTY medium supplemented with 100 µg/mL ampicillin and 70 µg/mL kanamycin), and grown, shaking overnight at 37°C. Finally, cultures were centrifuged (11,000 ×*g* for 30 min at 4°C), and the virion-containing supernatant was collected. The phage particles were precipitated by mixing four parts with one part of PEG/NaCl solution (20% (w/v) polyethylene glycol 6000, 2.5 M NaCl). After an incubation of 30 min on ice, the precipitated virus particles were harvested by centrifugation (2,200 ×*g* for 30 min at 4°C) and resuspended in PBS to a ﬁnal volume of 1 mL. The concentration of phage particles was determined by measuring the OD_260_ (an OD_260_ of 1 equals 3 × 10^10^ particles per mL). A total of 10^11^ phages (diluted in blocking buffer, see below) were used for solid-phase panning of the two libraries (Attitude and Cosimo) against both recombinant antigens used for immunization (PfCSP_FL_ and PfCSP_C_) and for in-solution panning of the Attitude library against PfCSP_FL-bio_. For solid-phase panning, the antigens (100 µg/mL in 0.1 M NaHCO_3_ at pH 8.2) were coated overnight at 4°C in the wells of a 96-well Nunc Maxisorb microtiter plate (Thermo Scientiﬁc). Protein binding sites in antigen-coated and non-coated wells were saturated for 2 h at room temperature with 200 µL 2% milk in PBS or Pierce Protein-Free T20 (PBS) blocking buffer (Thermo Scientific) as blocking reagents (alternated to avoid non-specific binders to buffer components), after which 100 µL of the phage suspension was added and incubated for 1 h. Phage particles were eluted by incubation in 100 µL of 100 mM triethylamine (TEA at pH 11.0) for 10 min, followed by neutralization with 100 µL 1 M Tris–HCl (pH 8.0). In between each step, the wells were washed 10–20 times with PBST (0.1% Tween 20 in PBS). For in-solution panning, PfCSP_FL-bio_ (25 mM) was mixed with the phages in 250 mL blocking buffer and incubated for 1 h. The following blocking buffers were alternated to avoid enrichment of non-specific binders: 1% BSA in TBST (50 mM Tris–HCl, 150 mM NaCl, 0.05% Tween 20, pH 7.5), 0.1% casein in TBST, and Pierce Protein-Free T20 (PBS) blocking buffer. Per panning round, 20 µL Pierce streptavidin magnetic beads (Thermo Scientific) was added to 1 mL TBST and subsequently blocked for 1 h in 1 mL blocking buffer, after which the beads were incubated with the panning mixture for 1 h, all while rotating at room temperature. The beads were subsequently washed seven times in 1 mL blocking buffer, followed by three times in 1 mL PBST, each wash performed while rotating the beads for 5 min. Bound phages were eluted and neutralized as described above. The magnetic beads were manipulated using a magnetic stand. Eluted phage particles were amplified by: (i) infecting 2 mL of fresh, exponentially growing *E. coli* TG1 cells (in LB medium) for 30 min at 37°C; (ii) diluting the culture with 8 mL 2xTY-Gluc-Amp medium and continuing incubating for 1 h at 37°C with shaking; (iii) super-infecting the cells with 10^7^ M13K07 helper phages for 30 min at room temperature; and (iv) pelleting the bacteria by centrifugation (800 *×g* for 10 min at 4°C) to inoculate 150 mL 2xTY-Amp-Kan medium, growing them overnight by shaking at 37°C. Panning of the immune libraries was performed for three (Cosimo) and four (Attitude) consecutive rounds on recombinant antigens. Enrichment of antigen-specific phages was investigated by infecting 90 µL exponentially growing *E. coli* TG1 cells (in LB medium) with 10 µL of a 10-fold dilution series (10^−1^–10^−7^ in PBS) of the eluted phages (from the antigen-coated and non-coated wells) in a round bottom 96-well plate for 30 min at 37°C, after which 10 µL per transduced cell culture was applied in a line on a square LB-agar-Gluc-Amp plate. The remaining cell cultures were plated on round LB-agar-Gluc-Amp plates to grow single colonies. All plates were incubated overnight at 37°C and subsequently stored at 4°C until further use.

*Identification of PfCSP-specific sdAbs*. Screening and selection of colonies carrying sdAb fragments specifically binding the target antigens were performed through an enzyme-linked immunosorbent assay (ELISA) on crude bacterial extracts (BEs). From the last two rounds of panning of the sdAb library on recombinant target antigens, 47 individual colonies were randomly selected per target antigen and grown in 100 µL of 2xTY-Gluc-Glyc-Amp medium (2xTY medium supplemented with 2% D-glucose, 10% glycerol, and 100 µg/mL ampicillin) in 96-well round bottom culture plates. After overnight growing at 37°C, 10 µL of each clone was used to inoculate 1 mL of 2xTY-Gluc-Amp medium (0.1% D-glucose) in 96 deep-well plates. After growing at 37°C for 4–5 h with aeration, the cultures were induced with 1 mM IPTG and incubated for another 4 h, after which cells were harvested by centrifugation (3,220 *×g* for 20 min at 4°C). The BEs containing the sdAbs were released through a freeze–thaw cycle, after which the cell pellets were resuspended in 100 µL PBS. Subsequently, the plates were centrifuged (3,000 ×*g* for 20 min at 4°C), and the BEs (supernatants) were collected into a new 96-well plate. In parallel, Nunc Maxisorb microtiter plates were coated (2.5 µg/mL antigen solution), blocked, and washed as described above. Non-coated wells were used as a negative control for each colony. Fivefold dilutions of each BE were loaded (100 µL) onto blocked coated and non-coated wells and incubated for 1 h at room temperature. The ELISA was detected by 1 h incubations at room temperature with 100 µL mouse anti-hemagglutinin mAb (anti-HA, BioLegend 16B12) and goat anti-mouse pAb conjugated with alkaline phosphatase (AP; Bethyl Laboratories A90-116AP), which were 2,000-fold diluted in PBS. The ELISA was developed with AP substrate (2 mg/mL 4-nitrophenyl phosphate disodium salt hexahydrate in 100 mM Tris–HCl, 10 mM NaCl, 50 mM MgCl_2_, pH 9.5), and absorption was measured at 405 nm. A true binder was considered when the signal ratio between coated/uncoated wells was ≥2.

*Recombinant production and purification of anti-PfCSP sdAbs*. Chemocompetent *E. coli* WK6 cells were heat-transformed with the pMECS-GG-sdAb plasmids as described above. Transformants were selected by plating the cell culture on LB-agar-Gluc-Amp plates. Finally, single colonies were picked to inoculate 10 mL 2xTY-Amp medium (2xTY medium supplemented with 100 µg/mL ampicillin) and grown overnight by shaking at 37°C. The next day, glycerol stocks were prepared by mixing 900 µL culture with 900 µL sterile 50% (v/v) glycerol and stored at −80°C. Pre-cultures of 10–25 mL were started by inoculating LB-Amp or 2xTY-Amp medium with a single colony from a fresh transformation or cell paste from an existing glycerol stock and grown overnight by shaking at 37°C. Main cultures of 0.25–1 L were started in baffled culture flasks by inoculating TB-Gluc-Amp medium (1.2% tryptone, 2.4% yeast extract, 17 mM KH_2_PO_4_, 72 mM K_2_HPO_4_, 0.4% glycerol, supplemented with 100 µg/mL ampicillin and 0.2% D-glucose) with a 200-fold dilution of the pre-culture and grown by shaking at 37°C to an OD_600_ of 0.6–0.9, at which the gene expression was induced with 1 mM IPTG, and the temperature was decreased to 28°C for overnight incubation. Cells were harvested by centrifugation (3,220 ×*g* for 30 min or 11,000 ×*g* for 10 min at 4°C), after which the supernatant was discarded. Cell pellets were subjected to an osmotic shock by: (i) resuspension in 15 mL TES buffer (200 mM Tris–HCl, 500 mM sucrose, 0.5 mM EDTA, pH 8.0) per pellet of 1 L culture and shaking at 4°C for 6 h; and (ii) addition of 30 mL TES/4 buffer (50 mM Tris–HCl, 125 mM sucrose, 0.125 mM EDTA, pH 8.0) per pellet of 1 L culture and shaking overnight at 4°C. The periplasmic extracts (PEs) were collected by centrifugation (3,220 ×*g* for 30 min at 4°C) and stored in 50 mL Falcon tubes at 4°C. This process was repeated once to collect two PEs per sdAb.

sdAbs were purified from the PEs by a two-step protocol consisting of IMAC and SEC. Approximately 1–2 mL HisPur Ni-NTA resin (Thermo Scientific) per sdAb was equilibrated in PBS, divided over the PE-containing tubes, and incubated for 1 h at room temperature while gently shaking or rotating. The extracts were poured into empty PD10 columns with filters (GE Healthcare), after which the resin was washed with 20–30 bed volumes (BVs) PBS. The sdAbs were eluted by adding 1 BV elution buffer (0.5 M imidazole in PBS) with an incubation period of 10 min prior to collecting 1 mL fractions while adding more elution buffer. Elution fractions containing the sdAbs were identified via UV spectrophotometry, pooled, and subjected to SEC on HiLoad 16/60 Superdex 75 or HiLoad 16/60 Superdex 200 columns pre-equilibrated in PBS or TBS (20 mM Tris–HCl, 150 mM NaCl, pH 7.5). Relevant SEC elution fractions were pooled, aliquoted, and stored at −20°C.

### ELISA

Nunc Maxisorb microtiter plates were coated (1 µg/mL antigen solution), blocked (5% milk in PBS), and washed as described above. Non-coated wells were used as a negative control for each antigen. Diluted sdAbs (100 µL at 1 µg/mL in blocking buffer) were loaded in triplicates onto blocked wells and incubated for 1 h at room temperature, followed by incubations with 2,000-fold dilutions (in blocking buffer) of mouse anti-HA mAb and goat anti-mouse pAb conjugated with HRP (Sigma A4416). The ELISA was developed with 1-Step Ultra TMB-ELISA substrate solution (100 µL; Thermo Scientific) for 15–30 min, after which development was stopped with 1 M H_2_SO_4_ (50 µL), and absorption was measured at 450 nm. For the serum ELISA, Pierce Protein-Free T20 (PBS) blocking buffer was used as the blocking agent, and threefold dilution series (10- to 7,290-fold) of the Attitude and Cosimo sera were prepared in PBS and probed for binding to the antigens. Detection was performed with 2,000-fold dilutions (in blocking buffer) of rabbit anti-camelid VHH mAb (GenScript 96A3F5) and goat anti-rabbit pAb conjugated with AP (Bethyl Laboratories A120-101AP) and developed with AP substrate after which absorption was measured at 405 nm.

### Heparin binding assays

All proteins were extensively dialyzed against HAC buffer A prior to preparing 100 µL samples of the sdAb:PfCSP_FL_ complexes at a molar ratio of 1.2:1 and incubated for 1 h at room temperature. Approximately 100 µL Heparin Sepharose HP (GE Healthcare) per complex was equilibrated in HAC buffer A and incubated with the protein samples for 1 h. Subsequently, the samples were transferred to Pierce spin cups with cellulose acetate 0.45 µm filters (Thermo Scientific) and centrifuged (1,200 ×*g* for 1 min; Eppendorf Centrifuge 5417R). After that, the resin was washed twice with 50 µL HAC buffer A and eluted in two rounds with 50 µL HAC buffer B. All samples were collected and analyzed by SDS-PAGE.

### Sporozoite binding and invasion assays

*Ethical statement*. All animal experiments were performed in compliance with the French and European regulations regarding the protection and care of laboratory mice and approved by the ethics committee with references MESR 01324, APAFIS #32422-2021071317049057 v2, and APAFIS #32989-2021091516594748 v1.

*Procurement of midgut and salivary gland SPZs*. Transgenic *P. berghei* ANKA SPZs expressing full-length CSP from *P. falciparum* 3D7 (PfCSP-PbSPZs) were used. A green fluorescent protein (GFP) was expressed in the parasite cytoplasm to serve as a reporter (39). Naive *Anopheles stephensi* (strain SDA 500) mosquitoes were reared according to standard procedures. One day prior to infection, mosquitoes were deprived of sucrose to increase the bite rate, after which they were infected by allowing them to feed on two–three infected RjOrl:Swiss mice per 200 female mosquitoes for 30 min. Subsequently, mosquitoes were maintained in a humid chamber at 21°C for 1 week, after which they were allowed to feed on naive RjOrl:Swiss mice. MG and SG SPZs were isolated 13–22 days after infection in 10–30 µL 1× Dulbecco’s PBS and kept on ice.

*SPZ-sdAb binding studies*. About 20,000–30,000 PfCSP-PbSPZs per sdAb were fixed in 2% formaldehyde for 20 min at room temperature. Subsequently, the SPZs were washed once with PBS, after which they were centrifuged (500 ×*g* for 10 min at 4°C). The supernatant was then removed, and the pelleted SPZs were resuspended in 1 mL PBS with 2% BSA and incubated on ice for 1 h. If necessary, the SPZs were further diluted in PBS with 1% BSA to obtain about 8,000 SPZs in 20 µL per reaction tube. Thereafter, 10 µL SPZs were mixed with 10 µL 20 µg/ml sdAb, and the reaction mixtures were incubated for 1 h on ice or 30 min at room temperature. Then, a 500-fold dilution of mouse anti-HA mAb conjugated with Alexa Fluor 647 tag (BioLegend, clone 16B12) was added and incubated for 30 min on ice or 15 min at room temperature. Finally, the samples were diluted with 200 µL PBS, and data were collected on a CytoFLEX S flow cytometer (Beckman Coulter). For fluorescence microscopy, 20 µL of the final samples was transferred to a µ-Slide 18 well coverslip and centrifuged for 5 min at 500 ×*g* prior to image acquisition with an inverted Axio Observer Z.1 microscope. A non-relevant sdAb targeting β-lactamase from *Bacillus cereus* 596H (sdAb-BcII10) (40) was used as a control.

*SPZ hepatocyte traversal and cell wounding assays*. Invasion assays were performed as previously reported (41). HepG2 cells were seeded on flat-bottom 96-well plates (40,000 cells per well) 1 day before the SPZ challenge in DMEM (Invitrogen) supplemented with 10% heat-inactivated FCS, 1× of non-essential amino acids (Sigma), and 2% of penicillin–streptomycin–neomycin antibiotic mixture (Sigma) at 37°C with 5% CO_2_. PfCSP–PbSPZ–sdAb samples were prepared as described above, after which 50 µL/well was transferred to the HepG2 cells. Tetramethylrhodamine dextran, lysine-fixable, 10,000 MW (DxRed at 1 mg/mL; Molecular Probes) was added to detect cell wounding. The plates were centrifuged (500 ×*g* for 5 min at 10°C) and subsequently incubated for 2 h at 37°C with 5% CO_2_ and 10% O_2_. Non-targeting sdAb and cytochalasin D (CytoD; 0.5 µM) were included as controls for SPZ invasion. Unchallenged HepG2 cells and manually scratched HepG2 cells were used as additional controls for cell wounding. The supernatants were transferred to another plate, and the HepG2 cells were washed twice with 50 µL/well PBS. The wash fractions were pooled with the former supernatant and corresponded to the non-adherent SPZ fraction. Subsequently, 50 µL/well trypsin was added to the HepG2 cells and incubated for 10 min at 37°C, and trypsinization was stopped by adding 150 µL/well PBS with 2% FCS. After homogenizing the solutions in the wells, they were filtered to collect the extracellular (adherent) and intracellular (invasion) SPZ fractions. Samples of 50 µL from both fractions were analyzed on a CytoFLEX S flow cytometer. DxRed${}^{+}$ and GFP${}^{+}$ HepG2 cells are considered as wounded and invaded cells, respectively; GFP${}^{+}$ and GFP${}^{-}$ SPZs are viable and dead parasites, respectively. At 44 h, intracellular development was assessed based on the number of GFP${}^{+}$ cells. Statistical significance was determined with a one-way analysis of variance with Holm–Šídák correction for multiple comparisons using GraphPad Prism 10 (see Table S1 in the Supplemental material).

### Crystallization, data collection and processing, and structure determination

The sdAb:PfCSP_C_ complexes were prepared at a molar ratio of 1.2:1 and incubated for 1 h at room temperature. Subsequently, the complexes were concentrated to 200 µL using Vivaspin 6 (3 k) concentrator devices (Sartorius) and purified by SEC using an ENrich SEC 70 10 × 300 column (Bio-Rad), pre-equilibrated in 20 mM Tris–HCl, 150 mM NaCl, and pH 7.5. Relevant elution fractions containing the complex were pooled and concentrated to 5–10 mg/mL using Vivaspin 500 (3 k) concentrator devices (Sartorius). Crystallization conditions were screened by the sitting drop method using the Mosquito Xtal3 robotic system (TTP Labtech) mixing 100 nL protein droplets with 100 nL mother liquor in 96 well plates. Commercial screens of Jena Bioscience (JBScreen Basic, JBScreen Classic, JBScreen Wizard, and JBScreen PEG salt) and Molecular Dimensions (ProPlex, PACT Premier, and JCSG Plus) were used for screening. The crystal plates were incubated at 20°C, and crystal growth was monitored manually using a Nikon SMZ 745T stereo microscope. The affinity tags of all proteins were retained for crystallization. Diffraction-quality crystals of sdAb1:PfCSP_C_ and sdAb9:PfCSP_C_ were obtained after 3 to 4 days in JBScreen Wizard (Jena Bioscience) condition no. F12 (30% PEG 8000, 100 mM imidazole pH 8.0, 200 mM NaCl) and JBScreen Classic 1–4 (Jena Bioscience) condition no. D12 (35% PEG 4000), respectively.

The sdAb1:PfCSP_C_ and sdAb9:PfCSP_C_ crystals were cryocooled in liquid nitrogen with the addition of 25 (v/v) glycerol and 30% (v/v) glycerol to the mother liquor as a cryoprotectant, respectively. Data sets were collected at the ESRF Synchrotron (Grenoble, France) on the ID30A-3 beamline. Both data sets were processed with XDSME (42, 43). The quality of the collected data sets was verified by close inspection of the XDS output files and through phenix.xtriage in the PHENIX package (44). Twinning tests were also performed by phenix.xtriage. Analysis of the unit cell contents was performed with the program MATTHEWS_COEF, which is part of the CCP4 package (45). The structures of sdAb1:PfCSP_C_ and sdAb9:PfCSP_C_ were determined by molecular replacement with PHASER-MR (46). The following search models were employed for molecular replacement: (i) one copy of the structure of PfCSP_C_ (chain A, PDB ID 3VDJ); and (ii) one copy of an AlphaFold2 (47, 48) model of the sdAb (of which the CDRs were removed due to poor pLDDT scores). For both data sets, the searches provided single solutions (top TFZ = 22.6 and top LLG = 995.328 for sdAb1:PfCSP_C_, top TFZ = 20.3 and top LLG = 549.409 for sdAb9:PfCSP_C_). For both structures, refinement cycles using the maximum likelihood target function cycles of phenix.refine (44) were alternated with manual building using Coot (49). The crystallographic data for the sdAb1:PfCSP_C_ and sdAb9:PfCSP_C_ structures are summarized in Table S2 in the Supplemental material and have been deposited in the PDB (PDB IDs: 9HZJ and 9HZK, respectively). Molecular graphics and analyses were performed with UCSF ChimeraX (50).

### AlphaFold-based structure prediction

The structural models of sdAb:PfCSP_C_ protein complexes were predicted using AlphaFold-Multimer (51). Twenty-five models were predicted per run, and the best models underwent a final relaxation step. The models were evaluated based on the following parameters: AlphaFold-Multimer model confidence (a weighted combination of the predicted template modeling (pTM) and interface predicted template modeling (ipTM) scores [51]: 0.8*ipTM + 0.2*pTM), the predicted aligned error (PAE) matrix (47), the local and global predicted local distance difference test (pLDDT) scores (47), the predicted DockQ (pDockQ) values (52), and the normalized discrete optimised protein energy (zDOPE) scores (53). Molecular graphics and analyses were performed with UCSF ChimeraX (50).

## RESULTS

### Camelid immunization with two different PfCSP constructs yields 12 sdAbs specifically targeting PfCSP’s αTSR domain

**Fig 1:**
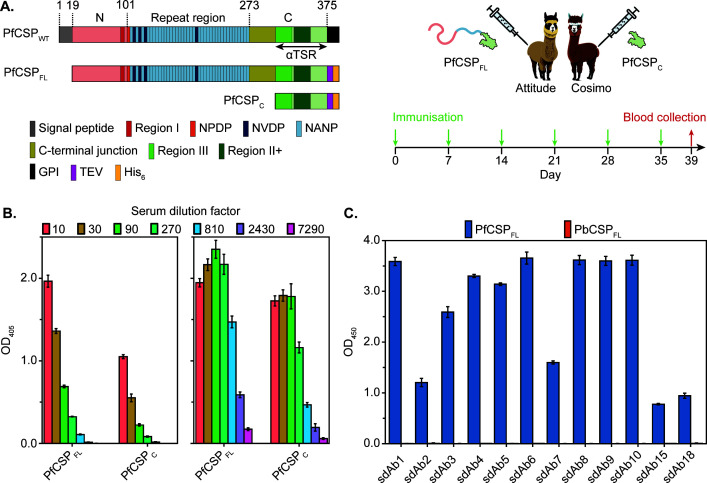
Generation of PfCSP-specific sdAbs. (A) Schematic overview of the wild-type (WT) PfCSP from *P. falciparum* NF54 and the recombinant protein constructs derived from it shown in the left panel. The start of each domain and the segment corresponding to the globular αTSR domain are indicated on top and below the WT schematic, respectively. A legend of the conserved regions, tandem repeats, and purification tags (TEV, tobacco etch virus protease cleavage site; His6, hexahistidine tag) is shown at the bottom. An overview of the alpaca immunization strategy used to generate two immune VHH libraries is provided in the right panel. (B) Serum ELISA. Identification of HCAbs in the sera of Attitude (left panel) and Cosimo (right panel) targeting recombinant PfCSP_FL_ and PfCSP_C_. (C) Cross-reactivity ELISA. The sdAbs raised against PfCSP were probed for binding to PfCSP_FL_ and PbCSP_FL_ (blue and orange bars, respectively). (B, C) The mean and standard deviation of triplicates for each condition are shown.

Two anti-PfCSP VHH libraries were generated through the immunization of two alpacas (Attitude and Cosimo) with distinct recombinant antigens (Fig. 1A): while Attitude was immunized with full-length PfCSP (PfCSP_FL_, devoid of its signal peptide and GPI anchor), Cosimo only received PfCSP_C_ (corresponding to the αTSR domain). Peripheral blood samples were collected 4 days after the last immunization. The induced immune responses against the antigens were assessed via serum ELISA, indicating that the sera of both animals contained PfCSP-specific HCAbs (Fig. 1B). Interestingly, significant binding signals are observed for PfCSP_C_ with both sera, indicating that a substantial HCAb subset in the Attitude serum targets conformational epitopes within the globular αTSR domain despite the presentation of the linear B-cell epitopes in PfCSP’s repeat region.

The two VHH libraries contained 8 × 10^7^ (Attitude) and 2 × 10^9^ (Cosimo) individual transformants, with an insert percentage of ∼100%. PfCSP-specific sdAbs were enriched by phage display using both solid-state and in-solution panning. The same seven VHH families were identified after both panning strategies (see Fig. S1 in the Supplemental material). Interestingly, all positive clones recognize both PfCSP_FL_ and PfCSP_C_, demonstrating that all sdAbs target epitopes within the αTSR domain. Eventually, 12 sdAbs (sdAb1-10 from Attitude; sdAb15 and sdAb18 from Cosimo) were selected for further characterization. Because CSP_C_ contains several regions that are relatively well conserved among *Plasmodium* species (e.g., regions III and II+), cross-reactivity of the sdAbs against recombinant full-length CSP from *P. berghei* (PbCSP_FL_) was assessed by ELISA (Fig. 1C). While clear binding can be observed to PfCSP_FL_, none of the sdAbs recognize PbCSP_FL_, indicating that key residues essential for the interaction are absent in PbCSP_FL_.

### The anti-PfCSP_C_ sdAbs do not interfere with heparin binding

**Fig 2:**
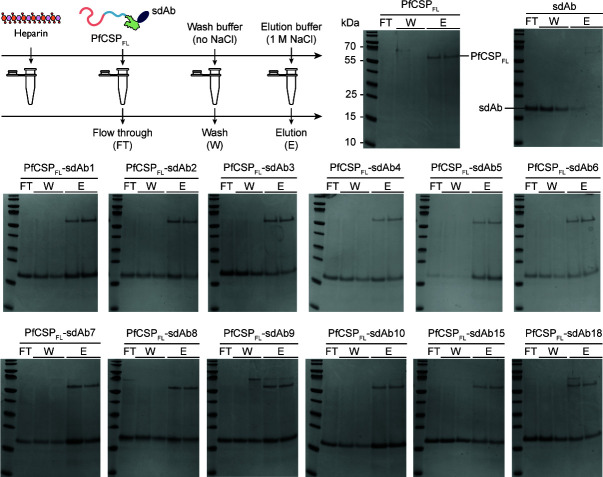
Heparin binding experiments with sdAb–PfCSP_FL_ samples. The experimental setup is schematically illustrated in the top left panel. All collected fractions were analyzed by SDS-PAGE. Unbound PfCSP_FL_ and sdAb were included as positive and negative controls, respectively.

CSP is essential to initiate the liver stage of the infection via its specific interaction with liver cell HS-HSPGs. Although PfCSP_N_ is presumed to be the main contributor to this interaction, a possible supporting role has been suggested for PfCSP_C_ (54). Therefore, the ability of the sdAbs to inhibit the interaction between PfCSP and heparin was investigated. Heparin is a structural analogue of HS-HSPGs that is frequently used in malaria research (18, 54–56). sdAb-PfCSP_FL_ complexes were prepared with a slight excess of sdAb to ensure complete antigen saturation and subsequently probed for binding to heparin in a spin cup filter setup (Fig. 2). After several washing steps, heparin-bound proteins were eluted with a high-salt buffer (1 M NaCl). The results were visualized by subjecting all collected fractions (flow through, FT; wash, W; elution, E) to SDS-PAGE. PfCSP_FL_ and one of the sdAbs were used as positive and negative controls, respectively. Sample analysis shows that the positive control only displays a protein band between the 55 and 70 kDa markers (expected for PfCSP_FL_) in the elution fractions, while the negative control only exhibits a protein band just above the 15 kDa marker (expected for a sdAb) in the flow through and wash fractions. A small and barely visible sdAb band is present in the first elution fraction as well, which is most probably due to the abundance in which the protein was used rather than a specific interaction with heparin. Hence, the controls indicate that PfCSP_FL_ is indeed able to interact with heparin, and that the possibility of the formation of a heparin-sdAb complex can be discarded. Analysis of the sdAb-PfCSP_FL_ samples shows that the sdAbs were present in all collected fractions, while PfCSP_FL_ can only be observed in the elution fractions. This demonstrates that the sdAbs are indeed able to bind PfCSP_FL_ but do not have the ability to inhibit its interaction with heparin. Hence, none of the sdAbs impair the PfCSP–heparin interaction.

### The anti-PfCSP_C_ sdAbs bind salivary gland SPZs but do not inhibit productive SPZ invasion

**Fig 3:**
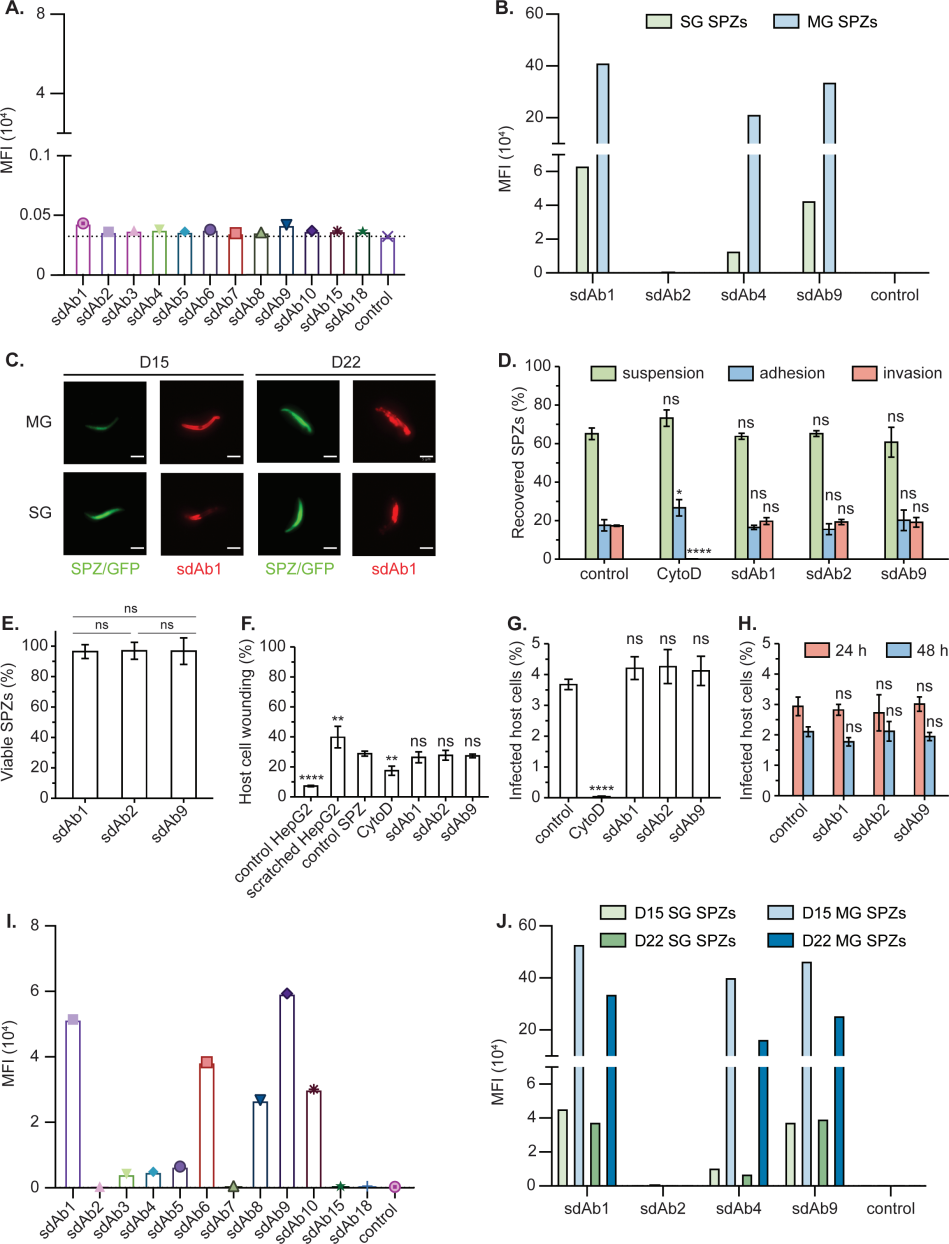
In vitro interaction studies between the anti-PfCSP sdAbs and transgenic PfCSP–PbSPZs. (A) Interaction screening of the sdAbs to 20-day-old SG SPZs on ice. PBS was used as a negative control. (B) Interaction studies of selected sdAbs to 15-day-old MG and SG SPZs at ambient temperature. sdAb-BcII10 was included as a negative control. (C) Fluorescence imaging of MG and SG SPZs after incubation with sdAb1 at ambient temperature. Scale bars (white lines): 5 µm. (D–G) Effect of the sdAbs on SPZ invasion of HepG2 cells evaluated 2 h after parasite addition. Here, we measured the recovery of SPZs present in the suspension, attached to, or inside the cells (D), percentage of viable SPZs compared to the control (E), percentage of wounded cells (F), and percentage of infected cells (G). sdAb-BcII10 and CytoD were included as negative and positive controls for invasion inhibition, respectively. (H) Effect of the sdAbs on the SPZ intracellular development. The percentage of parasite-infected HepG2 cells 24 and 48 h post-incubation with SPZs pre-incubated with the different sdAbs is shown. sdAb-BcII10 was included as a negative control. (I) Interaction screening of the sdAbs to 13-day-old MG SPZs on ice. PBS was included as a negative control. (J) sdAb binding to MG and SG SPZs with different maturation times (15-day-old vs. 22-day-old) at ambient temperature. sdAb-BcII10 was included as a negative control. (A, B and I, J) Binding is expressed as median fluorescence intensities (MFI). (D–H) The mean values with standard deviation of three independent measurements are shown. Statistical significance was determined by one-way analysis of variance with Holm–Šídák correction for multiple comparisons; *P ≤ 0.05, **P ≤ 0.01, ****P ¡ 0.0001, ns = not significant.

The ability of the sdAbs to recognize their target antigen on the surface of live SPZs was assessed using transgenic *P. berghei* SPZ expressing PfCSP (PfCSP–PbSPZs) (39). The results show that the recognition of SG SPZs heavily depends on the assay conditions. While the sdAbs displayed no to very poor interaction with SG SPZs at 10°C, better binding signals could be obtained for a selection of anti-PfCSP sdAbs (1, 4, and 9) at ambient temperature (Fig. 3, panels A to C). This discrepancy is most likely explained by the temperature’s influence on binding affinity and membrane fluidity, thereby affecting the sdAb°PfCSP interaction at the parasite surface. Nonetheless, the data indicate that some sdAbs can reach PfCSP_C_ on SG SPZs.

Next, the anti-PfCSP sdAbs 1, 2, and 9 were tested for their effect on the viability and invasion of PfCSP°PbSPZs *in vitro*. SPZs were pretreated with the sdAbs, after which their abilities to adhere to and invade HepG2 cells were assessed. Since SPZs rely on the actin–myosin motor for their migration and invasion of host cells (57), Cytochalasin D (CytoD, an inhibitor of actin polymerization) was used as a negative control for cell invasion. None of the tested anti-PfCSP sdAbs have an inhibitory effect on SPZ adhesion or invasion (Fig. 3D), nor do they affect SPZ viability (Fig. 3E). In addition, the sdAbs have no effect on SPZ cell traversal (Fig. 3F). Quantification of parasite-infected HepG2 cells 2 h post-initiation validates that there is indeed no difference between the SPZs treated with the control sdAb and the anti-PfCSP sdAbs (Fig. 3G). Finally, parasite liver stage development was monitored for 48 h, demonstrating that there is no sdAb-mediated inhibition (Fig. 3H). In conclusion, despite being able to reach their epitope on SG SPZs, the anti-PfCSP sdAbs do not confer protection against host cell infection and parasite development.

### The anti-PfCSP_C_ sdAbs display prominent binding to midgut sporozoites

Because CSP has been reported to adopt different conformations depending on the SPZ developmental stage within the mosquito (33), all sdAbs were also assayed for binding to MG SPZs. At 10°C, sdAbs 1, 6, 8, 9, and 10 display a clear interaction with MG SPZs, whereas the other sdAbs show weaker binding (sdAbs 3, 4, and 5) or no binding at all (sdAbs 7, 15, and 18) (Fig. 3I). Not surprisingly, the detection signals were larger at ambient temperature (recorded for sdAbs 1, 4, and 9) (Fig. 3, panels B and C). Clearly, these experiments demonstrate that these anti-PfCSP sdAbs bind MG SPZ more efficiently compared to SG SPZs. To investigate the influence of the SPZ maturation state on sdAb recognition, a similar experiment was performed with 15- and 22-day-old MG and SG SPZs (Fig. 3J). This reveals that binding depends on the SPZ developmental stage rather than SPZ maturation. Indeed, fluorescence imaging of fixed PfCSP–PbSPZs shows that sdAb1 more readily interacts with MG SPZs compared to SG SPZs, independent of their maturation state (Fig. 3E). Together, these results indicate that, while the epitopes targeted by these sdAbs are easily accessible on MG SPZs, they may be (partially) shielded or less accessible on SG SPZs.

### Most sdAbs target the α-epitope

To unravel the structural basis of PfCSP_C_ recognition by the identified anti-PfCSP sdAbs, the high-resolution structures of sdAb–PfCSP_C_ complexes were either experimentally determined through macromolecular X-ray crystallography (MX) or predicted using AlphaFold-Multimer.

**Fig 4:**
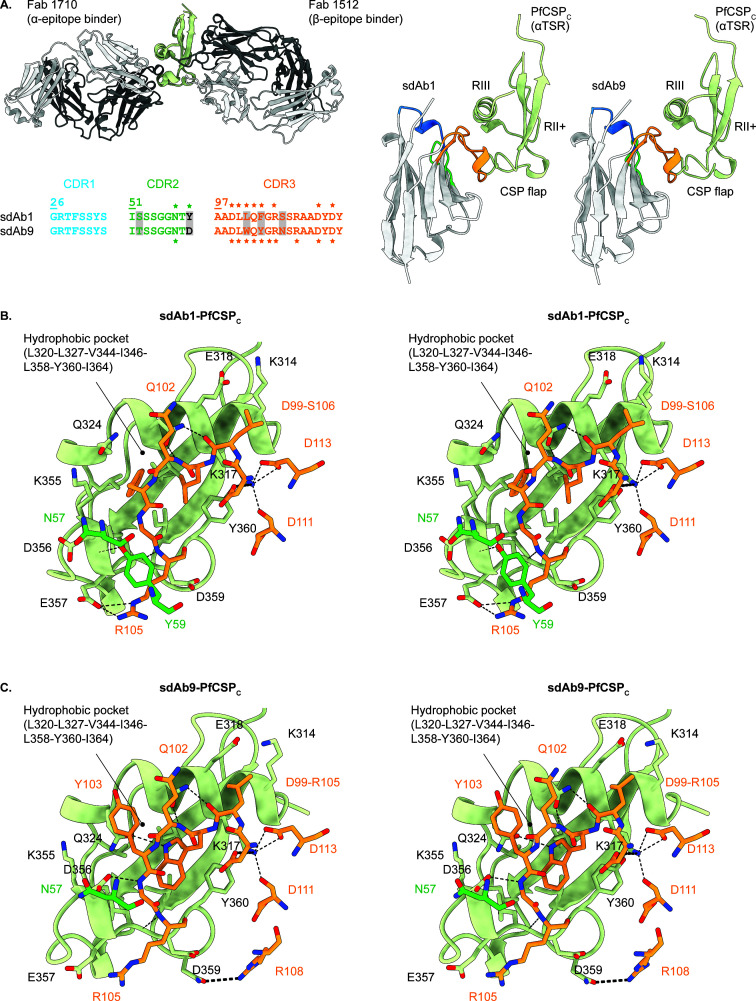
Crystal structures of sdAb1 and sdAb9 in complex with PfCSP_C_. (A) Cartoon representations of PfCSP_C_ (in light green) in complex with antibody fragments (in gray). The top left panel displays a superposition of the Fab1710-PfCSP_C_ (PDB ID 6B0S [30]) and Fab1512-PfCSP_C_ (PDB ID 7RXP [31]) crystal structures. The right panel shows the crystal structures of PfCSP_C_ bound by sdAb1 and sdAb9 (PDB IDs 9HZJ and 9HZK, respectively, this work). A sequence alignment of the CDRs from sdAb1 and sdAb9 is presented in the bottom left panel, with amino acid substitutions highlighted by gray boxes. The CDRs are indicated in different colors (CDR1, blue; CDR2, green; CDR3, orange), and the residues that are part of the paratope are marked by an asterisk (*). Stereo views of the sdAb1–PfCSP_C_ (B) and sdAb9–PfCSP_C_ (C) interactions. PfCSP_C_ is depicted in cartoon representation. For reasons of clarity, only the sdAb residues that are part of the paratope are shown and colored as in (A). All interacting residues are labeled and shown in stick representation. Hydrogen bonds and salt bridges are indicated by black dashed lines.

Crystallization trials were initiated for PfCSP_C_ complexed with sdAb1, 4, 6, and 9. Diffraction quality crystals were obtained for PfCSP_C_–sdAb1 and PfCSP_C_–sdAb9 complexes, and data were collected to ∼2.1 Å (see Table S2 in the Supplemental material). The crystal structures reveal that sdAb1 and 9 target the PfCSP_C_α-epitope (30, 31) (Fig. 4A). This epitope is composed of the Region III residues (adopting an α-helical structure), the flexible loop between the two antiparallel β-strands of the TSR fold (referred to as the CSP-flap), and the hydrophobic pocket between the α-helix and the CSP-flap. The paratopes of sdAb1 and 9 are largely similar, which is expected, as they belong to the same VHH family (Fig. 4A). The bulk of antigen contacts are provided by residues from their CDR3; however, N57 (CDR2) and Y59 (FR, only for sdAb1) also contribute to the interaction (Fig. 4, panels B and C for sdAb1 and sdAb9, respectively). The total buried surface areas of the PfCSP_C_–sdAb1 and PfCSP_C_–sdAb9 interfaces are 626 and 747 Å^2^, respectively, and detailed overviews of all contacts are provided in Table S3 and S4 in the Supplemental material. Noteworthy ‘interaction hot spots’ are sdAb1 amino acids L101 and F103 that are inserted into the hydrophobic pocket delineated by PfCSP residues L320, L327, L358, Y360, and I364. The sdAb1 hydrophobic residues L101 and F103 are substituted by W101 and Y103 in sdAb9. Here, W101 is inserted into the PfCSP_C_ hydrophobic pocket, but Y103 is oriented outwards. Three other important interactions are the salt bridges formed at either side of the α-epitope between sdAb1 R105 and PfCSP E357 (left side, only for sdAb1–PfCSP_C_) and sdAb D99/D113 and PfCSP K317 (right side, both complexes), which likely stabilize the hydrophobic interaction center.

In the absence of diffraction-quality crystals for the other sdAb–PfCSP_C_ complexes, we wondered whether AlphaFold-Multimer would be able to predict these structures. As a positive control, AlphaFold-Multimer was employed to predict the sdAb1–PfCSP_C_ and sdAb9–PfCSP_C_ complexes. We note that the predictions were highly accurate, displaying an almost perfect overlap with the experimentally determined crystal structures (Cα RMSDs of 0.99 and 1.08 Å for sdAb1–PfCSP_C_ and sdAb9–PfCSP_C_, respectively). Furthermore, both predicted complexes are characterized by very good to excellent values of various local and global validation metrics (pLDDT, zDOPE, pTM, PAE, pDockQ, and ipTM) that are defined and visualized in the legend of Fig. S2 in the supplemental material. Having validated the performance of AlphaFold-Multimer for sdAbs 1 and 9, it was employed to predict the complexes for the other sdAb–PfCSP_C_ complexes. Reliable models could be obtained for all sdAbs, except for sdAb2, 4, and 8. A comparison with the sdAb1–PfCSP_C_ and sdAb9–PfCSP_C_ complex structures reveals that the sdAbs retrieved in this study are highly likely to be α-epitope binders.

## DISCUSSION

Significant progress has been made in reducing malaria-endemic areas primarily by targeting the mosquito vector (58). While vector control remains a cornerstone of current malaria control programs, our understanding of parasite biology within the mosquito host remains limited. A notable example is the SPZ’s main surface antigen: while CSP has been shown to play a crucial role during SPZ development in the mosquito (9), many molecular details remain largely unexplored. Here, we identified 12 sdAbs targeting PfCSP by phage display and panning of two immune VHH libraries to investigate the antigen’s structure–function relationship. Regardless of the immune VHH library (Attitude vs. Cosimo) or the employed immunization strategy (PfCSP_FL_ vs. PfCSP_C_), all sdAbs target the globular αTSR domain. This is intriguing since most conventional anti-PfCSP Abs raised in humans and mice recognize repeat region epitopes (20, 22, 59, 60), and PfCSP_C_-targeting Abs are considered rare (30–32). HCAbs possess longer CDR loops (particularly CDR3) to maintain a sufficiently large paratope surface area for effective antigen recognition and compensate for the absence of a light chain (36). Therefore, VHHs possess unique paratope features that are usually not found in conventional Abs, giving them a preference for binding conformational epitopes (61–63), which may provide an explanation for our findings.

High-resolution structure determination through experimental methods (MX) or machine learning-based prediction (AlphaFold-Multimer) reveals that most sdAbs recognize the PfCSP_C_ α-epitope. For the sdAb1–PfCSP_C_ complex, the experimentally determined crystal structure further validates results recently obtained through a combination of molecular modeling and mass spectrometry (64). The recognition of PfCSP by the sdAbs is highly specific, as no binding to PbCSP could be observed. This is consistent with the literature on anti-PfCSP_C_ conventional Abs, which reports that cross-reactivity to PfCSP_C_ from different PfSPZ field isolates depends on β-epitope (and not α-epitope) characteristics (31). This can be attributed to the polymorphic nature of the Th2R (Region III) and Th3R (CSP-flap) regions constituting the α-epitope (65), while the β-epitope contains the more conserved regions II+ (TSR strands 1 and 2) and CS.T3 (TSR strand 3) (31, 66). As evidenced by heparin binding assays and functional experiments with live SG SPZs, the sdAbs studied in this work do not impede SPZ adhesion, traversal, and invasion of hepatocytes, nor its intracellular development. This is consistent with previous work reporting that α-epitope targeting Abs are poorly or non-protective (30). However, the underlying molecular mechanisms for these observations remain unclear. Three hypotheses could be presented: the targeted epitopes are (i) accessible but inherently non-protective, (ii) inaccessible to bulky, conventional Abs due to the densely packed CSP-covered parasite surface, or (iii) inaccessible due to the CSP conformation on the SG SPZ surface. Arguments for the latter two hypotheses are provided by the observation that both α- and β-epitope targeting Abs inefficiently bind SG SPZs (30–32). In contrast, due to their smaller size, sdAbs are known for their ability to easily penetrate densely coated antigen-crowded parasite surfaces and bind epitopes, which are inaccessible to conventional Abs (67, 68). Hence, while the second hypothesis cannot be excluded based on the data presented here, the sdAbs are likely to be able to infiltrate the densely packed CSP-covered parasite surface. However, the results clearly indicate that staining of SG SPZs using these sdAbs is comparable to that obtained through the use of mAbs (69).

**Fig 5:**
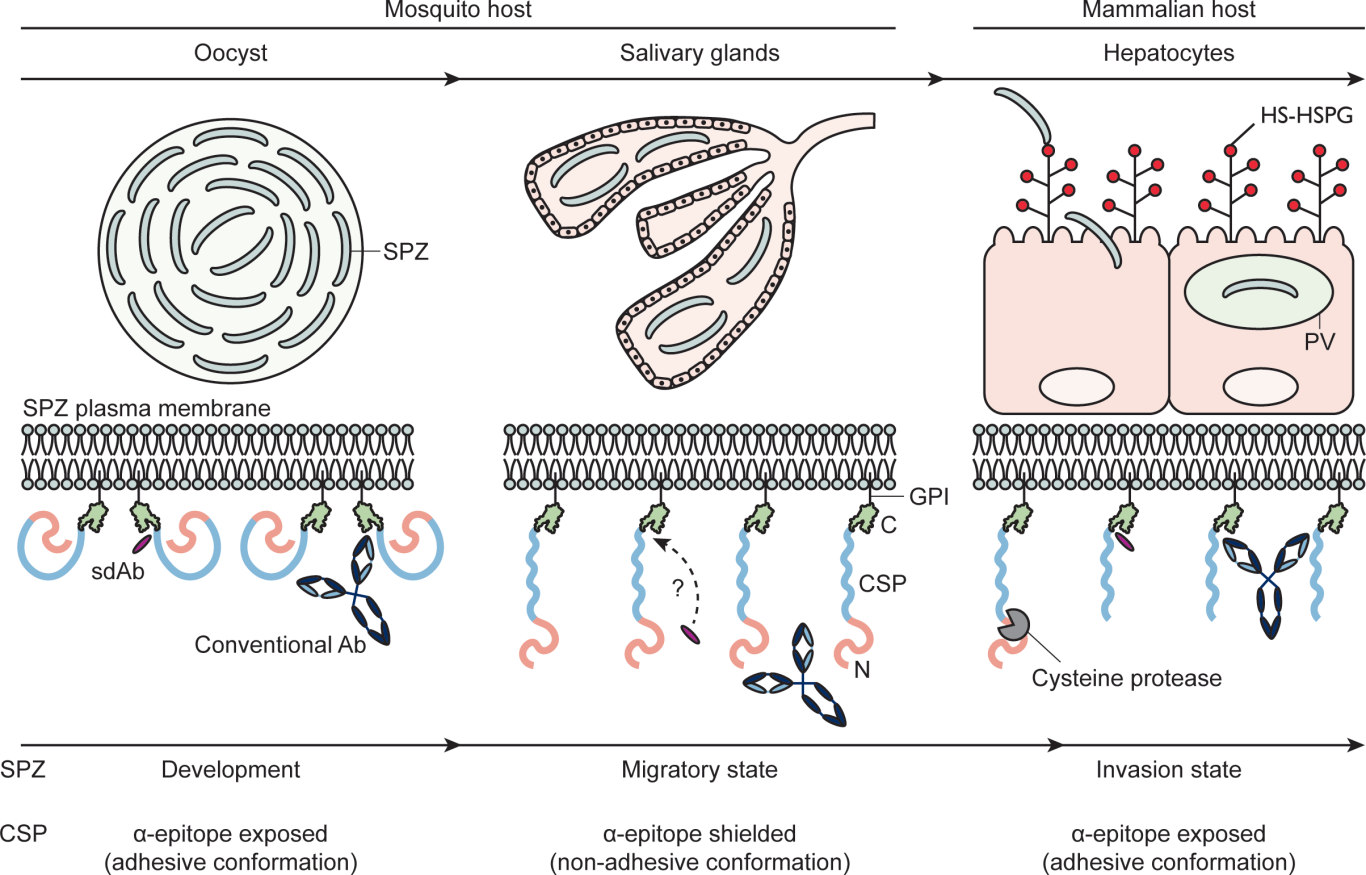
CSP is subject to conformational changes during SPZ development in the mosquito and its journey to the mammalian liver. CSP’s α-epitope is exposed during SPZ development in the mosquito midgut (adhesive conformation). Upon release from the oocyst, the SPZs transition to a migratory state and travel to the salivary glands. This transition is marked by a conformational change of CSP, rendering the α-epitope inaccessible to Abs and fragments thereof (non-adhesive form). sdAbs are expected to display better penetration properties due to their smaller size. Following deposition into the mammalian skin, the SPZs migrate to the liver, where they are primed for hepatocyte invasion through the interaction between CSP and highly sulfated heparan sulphate proteoglycans (HS-HSPGs). This interaction triggers the proteolytic cleavage of CSP by a cysteine protease, causing it to revert to its adhesive conformation and expose its α-epitope. Consequently, the SPZ switches to its invasion state, allowing it to invade a final hepatocyte and settle within a parasitophorous vacuole (PV) for its further development.

Finally, the most intriguing result presented in this work is perhaps the observation that, in comparison to SG SPZ, the α-epitope targeting sdAbs readily recognize MG SPZs. The observed difference in binding signals suggests that the accessibility of the α-epitope changes during SPZ development in the mosquito, which in turn supports a model for conformational change by PfCSP at the parasite surface (33). This could be an intrinsic property of the surface antigen and/or could be induced by a yet unknown mosquito factor. We propose that our findings further refine this model (Fig. 5): the α-epitope is exposed and accessible on the surface of MG SPZs (CSP adhesive form) but shielded and inaccessible to large molecules (e.g., Abs) on SG SPZs (CSP non-adhesive form). These conformations are closely linked to SPZ migration/invasion, as CSP takes its adhesive form during SPZ development in the oocyst and hepatocyte invasion, while the non-adhesive conformation is adopted during SPZ migration from the mosquito midgut to the mammalian liver. Further research on the exact timing of the transition from the adhesive to the non-adhesive conformation in the mosquito will enable further fine-tuning of the model and provide novel insights into parasite biology inside the mosquito host.

## Data Availability

The sdAb1–PfCSP_C_ and sdAb9–PfCSP_C_ crystal structures have been deposited in the PDB (accession codes 9HZJ and 9HZK). All other data are contained within this article or in the Supplemental material.

## SUPPLEMENTAL MATERIAL

Supplemental material iai.00081-25-s0001.pdf Table S1 to S4; Fig. S1 and S2.

ASM does not own the copyrights to Supplemental Material that may be linked to, or accessed through, an article. The authors have granted ASM a non-exclusive, world-wide license to publish the Supplemental Material files. Please contact the corresponding author directly for reuse.
