# Supplementary material for: Evidence for a model of conformational change by the Plasmodium falciparum circumsporozoite protein during sporozoite development in the mosquito host through the use of camelid single-domain antibodies
Source: Infect Immun. 2025 Apr 28;93(6):e00081-25. doi: 10.1128/iai.00081-25 (PMC12150756; doi:10.1128/iai.00081-25)
Supplement: Supplemental material — Table S1 to S4; Fig. S1 and S2. [file iai.00081-25-s0001.pdf]

Supplemental material

TABLE S1. Statistics of the SPZ hepatocyte traversal and invasion assays. Statistical significance was determined with a one-way ANOVA with Holm–Šídák correction for multiple comparisons using GraphPad Prism 10.

SPZ recovery (Fig 3D)	*P (suspension)*	*P (adhesion)*	*P (invasion)*
control vs. CytoD	0.1593	0.0437	<0.0001
control vs. sdAb1	0.9194	0.7772	0.2171
control vs. sdAb2	0.9793	0.7772	0.27
control vs. sdAb9	0.5593	0.7772	0.27
SPZ viability (Fig 3E)	*P*		
sdAb1 vs. sdAb2	0.9997		
sdAb1 vs. sdAb9	0.9997		
sdAb2 vs. sdAb9	0.9997		
Host cell wounding (Fig 3F)	*P*		
control SPZ vs. control HepG2	<0.0001		
control SPZ vs. scratched HepG2	0.008		
control SPZ vs. CytoD	0.0078		
control SPZ vs. sdAb1	0.7981		
control SPZ vs. sdAb2	0.8494		
control SPZ vs. sdAb9	0.8494		
Infected host cells (Fig 3G)	*P*		
control vs. CytoD	<0.0001		
control vs. sdAb1	0.235		
control vs. sdAb2	0.235		
control vs. sdAb9	0.235		
SPZ intracellular development (Fig 3H)	*P (24 h)*	*P (48 h)*	
control vs. sdAb1	0.9184	0.2231	
control vs. sdAb2	0.8715	0.9382	
control vs. sdAb9	0.9184	0.5974	

TABLE S2. X-ray diffraction data collection and refinement statistics. Statistics for the highest resolution shell are shown in parentheses.

	sdAb1:PfCSP$_C$	sdAb9:PfCSP$_C$
Data collection statistics		
Wavelength (Å)	0. 967697	0.967697
Resolution range (Å)	34.82-2.06 (2.27-2.06)	25.46-2.07 (2.28-2.07)
Space group	P212121	P32
a, b, c (Å)	37.19, 55.17, 89.78	53.74, 53.74, 60.84
alpha, beta, gamma (°)	90, 90, 90	90, 90, 120
Mosaicity (°)	0.086	0.202
Total number of measured reflections	76487 (18615)	43330 (11046)
Unique reflections	21593 (5376)	23186 (5814)
Multiplicity	3.5 (3.5)	1.9 (1.9)
Completeness (%)	98.31 (97.85)	99.57 (99.80)
<I\σ(I)>	6.92 (0.80)	5.36 (0.68)
Wilson B-factor (Å^2)	40.27	41.77
R_{merge} (%)	10.2 (108.0)	8.6 (89.9)
R_{meas} (%)	12.0 (128.2)	11.6 (121.1)
R_{pim} (%)	6.29 (68.41)	7.80 (80.52)
$CC_{1/2}$ (%)	99.3 (65.4)	99.30 (37.4)
A.U. contains	1 complex	1 complex
Refinement statistics		
CC*	0.998 (0.889)	0.998 (0.738)
R_{work} (%)	19.51 (39.48)	18.41 (37.60)
R_{free} (%)	24.24 (43.73)	22.61 (39.45)
Number of non-hydrogen atoms	1625	1646
macromolecules	1464	1475
ligands	37	81
solvent	124	90
Protein residues	190	192
RMS bond lengths (Å)	0.006	0.007
bond angles (°)	0.82	0.86
Ramachandran favoured (%)	97.85	98.94
allowed (%)	2.15	1.06
outliers (%)	0	0
Rotamer outliers (%)	0	0.63
Clashscore	1.35	2.60
Average B-factor (Å^2)	37.24	41.05
macromolecules (Å^2)	36.61	39.53
ligands (Å^2)	48.06	64.72
solvent (Å^2)	41.40	44.57

TABLE S3. List of interactions between sdAb1 and $PfCSP_C$.

sdAb1			$PfCSP_C$		
Residue	Region	Group	Residue	Group	Interaction (distance Å)
N57	CDR2	side chain	D356	side chain	Van der Waals
Y59	FR	side chain OH	D356	backbone CO	H-bond (2.72)
D99	CDR3	side chain	K317	side chain	electrostatic (2.84)
L100	CDR3	side chain	K314	side chain	Van der Waals
		side chain	K317	side chain	Van der Waals
		backbone CO	N321	side chain NH	H-bond (2.93)
L101	CDR3	side chain	K317	side chain	Van der Waals
		side chain	L320	side chain	hydrophobic
		side chain	Y360	side chain	hydrophobic
Q102	CDR3	backbone NH	N321	side chain CO	H-bond (2.88)
F103	CDR3	side chain	N321	side chain	Van der Waals
		side chain	Q324	side chain	Van der Waals
		side chain	L327	side chain	hydrophobic
		side chain	K355	side chain	Van der Waals
R105	CDR3	side chain	E357	side chain	electrostatic (2.81)
		backbone NH	L358	backbone CO	H-bond (2.78)
		side chain	D359	side chain	Van der Waals
		backbone CO	Y360	backbone NH	H-bond (2.93)
D111	CDR3	backbone CO	K317	side chain NH	H-bond (2.75)
		side chain	Y360	side chain	Van der Waals
		side chain	A361	side chain	Van der Waals
D113	CDR3	side chain	K317	side chain	electrostatic (2.80)

TABLE S4. List of interactions between sdAb9 and PfCSPc.

sdAb9			PfCSPc		
Residue	Region	Group	Residue	Group	Interaction (distance Å)
N57	CDR2	side chain NH	D356	side chain CO	H-bond (2.72)
D99	CDR3	side chain	K317	side chain	electrostatic (2.72)
L100	CDR3	side chain	K314	side chain	Van der Waals
		side chain	K317	side chain	Van der Waals
		side chain	E318	side chain	Van der Waals
		backbone CO	N321	side chain NH	H-bond (2.89)
W101	CDR3	side chain	K317	side chain	Van der Waals
		side chain	L320	side chain	hydrophobic
		side chain	N321	side chain	Van der Waals
		side chain	Y360	side chain	hydrophobic
		side chain NH	Q324	side chain CO	H-bond (3.47)
Q102	CDR3	backbone NH	N321	side chain CO	H-bond (2.98)
Y103	CDR3	side chain	N321	side chain	Van der Waals
		side chain	Q324	side chain	Van der Waals
		side chain	D356	side chain	Van der Waals
G104	CDR3	backbone NH	D356	side chain CO	H-bond (2.99)
R105	CDR3	side chain	E357	side chain	Van der Waals
		backbone NH	L358	backbone CO	H-bond (2.74)
		side chain	D359	side chain	Van der Waals
		backbone CO	Y360	backbone NH	H-bond (2.84)
R108	CDR3	side chain	A361	side chain	Van der Waals
		side chain NH	N362	sidechain NH	H-bond (3.58)
D111	CDR3	backbone CO	K317	side chain NH	H-bond (2.67)
		side chain	Y360	side chain	Van der Waals
D113	CDR3	side chain	K317	side chain	electrostatic (3.03)

Family	sdAb			CDR1			CDR2			
Family 1	sdAb1	QVQLQESGGG	LVQAGGSLRL	SCAAS**GRTFS**	**SYS**MGWFRLV	PGKEREFVAR	**ISSSGGNT**YY	ADSVRGRFTI	SRDNAKNTVY	LQMNSLKPED
	sdAb3	QVQLQESGGG	LVQAGGSLRL	SCAAS**GRTFT**	**EYS**MGWFRQA	PGKEREFVAR	**ISWSGGST**YY	ADSVKGRFTI	SRDNAENTVY	LQMTSLKPED
	sdAb7	QVQLQESGGG	VVQTGGSLRL	SCAAS**VRTFS**	**NYA**LGWFRQA	PGKEREFVAR	**ISSSGGNT**YY	ADSVRGRFTI	SRDNAKNTVY	LQMNSLKPED
	sdAb9	QVQLQESGGG	LVQAGGSLRL	SCAAS**GRTFS**	**SYS**MGWFRQV	PGKEREFVAR	**ITSSGGNT**DY	ADSAKGRFTI	SRDNAKNTVY	LQMSTLKPED
	sdAb18	QVQLQESGGG	LVQPGGSLNL	SCTA-**GVSLD**	**YYA**IGWFRQA	PGKEREFVAR	**ISSSGGNT**YY	ADSVRGRFTI	SRDNAKNTVY	LQMNSLKPED
Family 2	sdAb2	QVQLQESGGG	LVQFGGSLRL	SCAAS**GFTFS**	**SAN**MSWVRQA	PGKGLEWVST	**ISPGGGTT**SY	AGSVKGRFTI	SRDNAKNTLY	LQMNALKPED
Family 3	sdAb4	QVQLQESGGG	LVQVGGSLRL	SCAAS**GRTFS**	**SYT**MAWFRQA	PGKEREFVAA	**ISRSGSNT**YY	ADSVKGRFTI	SRDNAKNTVY	LQMSSLYPED
Family 4	sdAb5	QVQLQESGGG	LVQAGGSLRL	SCAAS**GRTFG**	**SYV**MGWFRQA	PGKEREFVAA	**ISRSGGTT**YY	ADSVKGRFAI	SRDNAKNTVF	LQMNSLKPED
Family 5	sdAb6	QVQLQESGGG	LVQAGGSLRV	SCVGS**GRAFS**	**RYS**MGWFRQF	PGKEREFVAC	**ISSSGGAI**DY	ADSVKGRFTI	SRDNAKNAVY	LQMNSLKPDD
Family 6	sdAb8	QVQLQESGGG	LVQAGGSLRL	SCAAS**GRTVS**	**SYT**MGWFRQA	PGKERSFVAA	**SLRSGGST**YY	SDSVKGRFTI	SRDNAKNTVY	LQMNSLRPED
Family 7	sdAb10	QVQLQESGGG	LVQPGGSLRL	SCAAS**GRTFS**	**SYT**MGWFRQA	PGKERELVAA	**ISRSGLNT**DY	ADSVKGRFTI	SRDNAKNTVY	LQMNTLKPED
	sdAb15	QVQLQESGGG	LVQAGGSLRL	SCVAT**GRPFD**	**DYT**VAWFRQA	PGKERDFVAA	**ISRSGVST**YY	QDDVKGRFTI	SRDNARNTAY	LSMNSLKPED

Family	sdAb		CDR3				
Family 1	sdAb1	TAVYYC**AAD-**	**-LLQFGRSSR**	**AADYDY**WGQG	TQVTVSSAAA	YPYDVPDYGS	HHHHHH
	sdAb3	TAVYYC**AADQ**	**--LQFGRNSR**	**AADYDD**WGQG	TQVTVSSAAA	YPYDVPDYGS	HHHHHH
	sdAb7	TAVYYC**AAD-**	**-LLQFGRSSR**	**AADYDY**WGQG	TQVTVSSAAA	YPYDVPDYGS	HHHHHH
	sdAb9	TAVYYC**AAD-**	**-LWQYGRNSR**	**AADYDY**WGQG	TQVTVSSAAA	YPYDVPDYGS	HHHHHH
	sdAb18	TAVYYC**AAD-**	**-LLQFGRSSR**	**AADYDY**WGQG	TQVTVSSAAA	YPYDVPDYGS	HHHHHH
Family 2	sdAb2	TAVYYC**ATG-**	**-------GPS**	**SQESVY**RGQG	TQVTVSSAAA	YPYDVPDYGS	HHHHHH
Family 3	sdAb4	TAVYTC**AARD**	**AILLL----T**	**TTHYDY**WGQG	TQVTVSSAAA	YPYDVPDYGS	HHHHHH
Family 4	sdAb5	TAVYYC**AATT**	**TISMLVVLTT**	**TPKYDY**WGQG	TQVTVSSAAA	YPYDVPDYGS	HHHHHH
Family 5	sdAb6	TAVYYC**AADQ**	**FRIST---VP**	**MGVIDY**WGQG	TQVTVSSAAA	YPYDVPDYGS	HHHHHH
Family 6	sdAb8	TAVYYC**AADE**	**QL-AYNINYN**	**YRNYDY**WGQG	TQVTVSSAAA	YPYDVPDYGS	HHHHHH
Family 7	sdAb10	TAVYYC**AARS**	**TIRLL----T**	**DPSYDY**WGQG	TQVTVSSAAA	YPYDVPDYGS	HHHHHH
	sdAb15	TAVYYC**AARS**	**TIRLL----T**	**DPSYDY**WGQG	TQVTVSSAAA	YPYDVPDYGS	HHHHHH

FIG S1. Amino acid sequence alignment of the selected anti-PfCSP sdAbs. The sequences are grouped by VHH family and the CDRs are highlighted in different colours (CDR1, blue; CDR2, green; CDR3, orange).

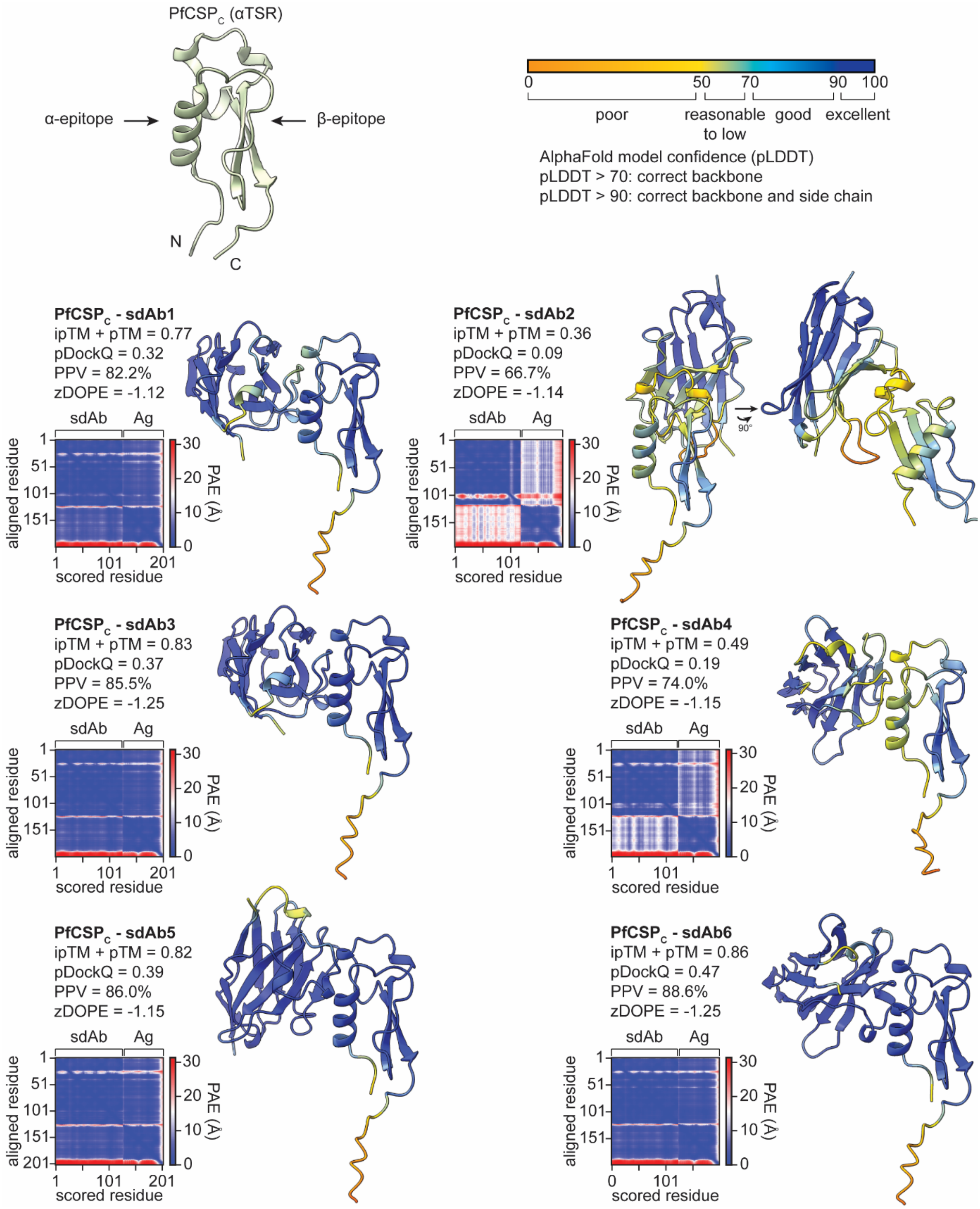

PfCSPc (αTSR)
α-epitope
β-epitope
N
C
0
50
70
90
100
poor
reasonable to low
good
excellent
AlphaFold model confidence (pLDDT)
pLDDT > 70: correct backbone
pLDDT > 90: correct backbone and side chain
PfCSPc - sdAb1
ipTM + pTM = 0.77
pDockQ = 0.32
PPV = 82.2%
zDOPE = -1.12
PfCSPc - sdAb2
ipTM + pTM = 0.36
pDockQ = 0.09
PPV = 66.7%
zDOPE = -1.14
90°
PfCSPc - sdAb3
ipTM + pTM = 0.83
pDockQ = 0.37
PPV = 85.5%
zDOPE = -1.25
PfCSPc - sdAb4
ipTM + pTM = 0.49
pDockQ = 0.19
PPV = 74.0%
zDOPE = -1.15
PfCSPc - sdAb5
ipTM + pTM = 0.82
pDockQ = 0.39
PPV = 86.0%
zDOPE = -1.15
PfCSPc - sdAb6
ipTM + pTM = 0.86
pDockQ = 0.47
PPV = 88.6%
zDOPE = -1.25
sdAb
Ag
aligned residue
scored residue
PAE (Å)

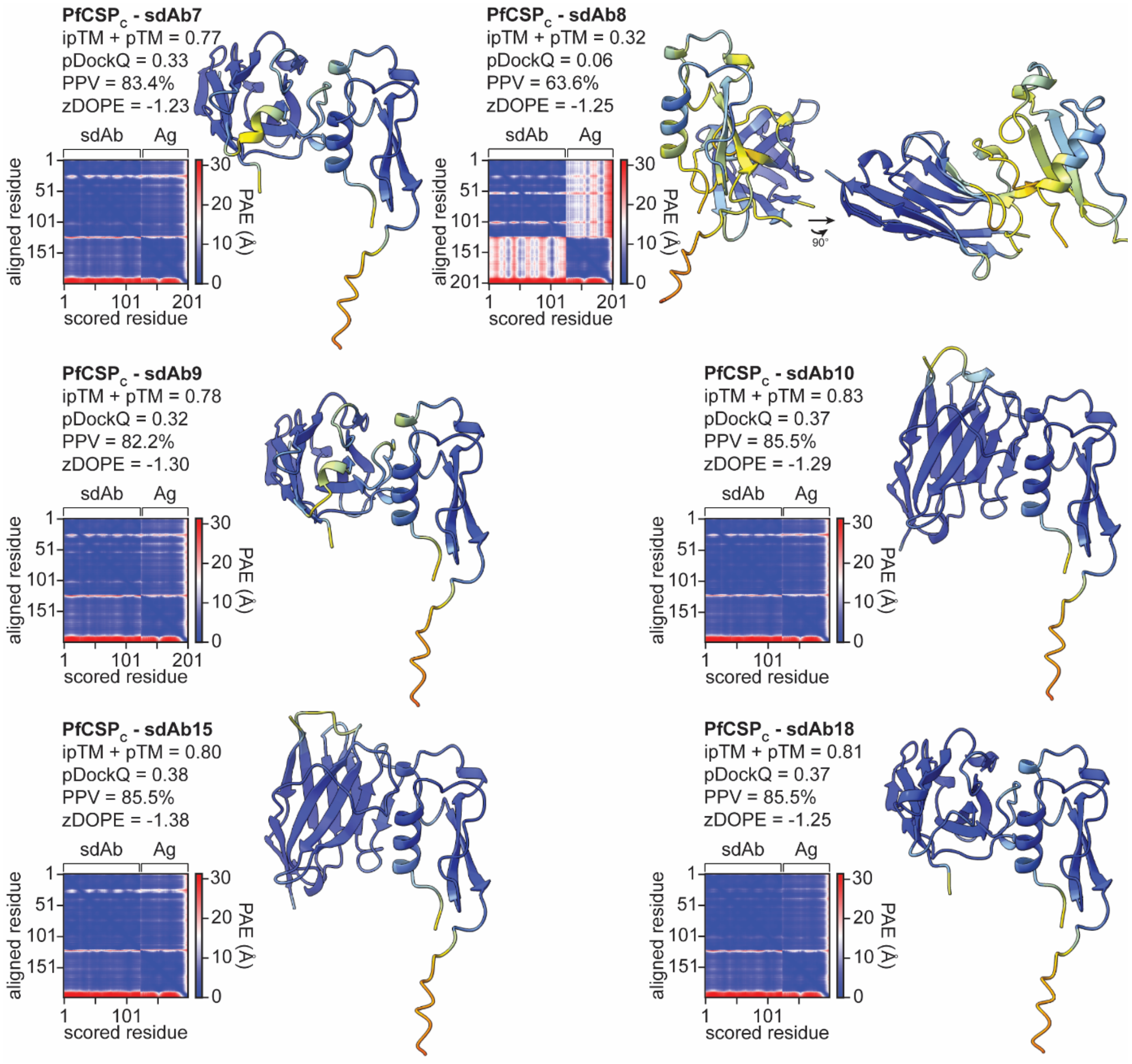

FIG S2. AlphaFold-Multimer models of the sdAb:PfCSP$_C$ complexes. The previously identified α- and β-epitopes are indicated on the crystal structure of PfCSP$_C$ (PDB ID: 3VDJ) in the top left panel. The AlphaFold structural models are colour coded according to their pLDDT scores (legend in the top right panel) and accompanied by their confidence metrics and PAE plots.
